# Tapping into natural history collections to assess latitudinal gradients of parasite diversity

**DOI:** 10.1017/S0031182023000458

**Published:** 2023-07

**Authors:** Sebastian Botero-Cañola, Scott L. Gardner

**Affiliations:** 1 H.W. Manter Laboratory of Parasitology, University of Nebraska State Museum, University of Nebraska - Lincoln, Lincoln, NE, USA; 2 Wisely Lab, University of Florida, Gainesville, FL, USA

**Keywords:** climate, compound community, helminth, host, Nearctic, parasitology collections

## Abstract

Parasites are key components of the biosphere not only due to their huge diversity, but also because they exert important influences on ecological processes. Nevertheless, we lack an understanding of the biogeographical patterns of parasite diversity. Here, we tap into the potential of biodiversity collections for understanding parasite biogeography. We assess species richness of supracommunities of helminth parasites infecting mammal assemblages in the Nearctic, and describe its relation to latitude, climate, host diversity, and land area. We compiled data from parasitology collections and assessed parasite diversity in Nearctic ecoregions for the entire parasite supracommunity of mammals in each ecoregion, as well as separately from carnivores and rodents to explore the effect of host taxonomic resolution on observed patterns. For carnivores, we found evidence of a negative latitudinal gradient, while parasites of rodents displayed no clear pattern. We found that parasite diversity was positively correlated with mean annual temperature and negatively correlated with seasonal precipitation. Parasite richness shows a diversity peak at intermediate host richness values and in carnivores correlates with temperature and seasonal precipitation. Rodent parasite diversity did not correlate with explored factors. Other researchers are encouraged to use parasitology collections to continue exploring patterns of parasite biogeography and macroecology.

## Graphical abstract

**Figure:**
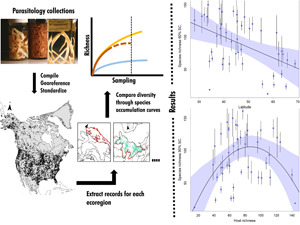

## Introduction

Parasites comprise a large portion of the world's biodiversity, with estimates of 40% to more than 50% of extant species having a parasitic lifestyle (Dobson *et al*., 2008; Larsen *et al*., 2017). Despite the ubiquity, diversity and ecological importance of the parasitic lifestyle (Thomas *et al*., 2005; Hudson *et al*., 2006; Lafferty *et al*., 2006; Sato *et al*., 2012; Larsen *et al*., 2017; Meyer *et al*., 2022), the understanding of both macroecological and biogeographical patterns of parasite diversity and their drivers lags behind that of free-living organisms (Carlson *et al*., 2020). Latitudinal gradients of diversity, one of the most explored and best described biogeographical patterns in free-living organisms (Willig *et al*., 2003), still present significant challenges from a parasitological perspective. Despite considerable research in recent decades (Kamiya *et al*., 2014; Preisser, 2019; Johnson and Haas, 2021; Martins *et al*., 2021; Preisser *et al*., 2022), the emergence of a general description of latitudinal variation in parasite species richness is still elusive. Currently and in the past, the detected patterns for this aspect of parasite biogeography have been dependent on the host and parasite taxa, as well as methodological approach (Preisser, 2019).

Mammals present the opportunity and necessity to study their parasitic fauna biogeographical patterns given the accumulated research effort, understanding of host diversity patterns and their potential to harbour zoonotic parasites (Kaufman and Willig, 1998; Schipper *et al*., 2008; Han *et al*., 2016). Studies focusing on parasite richness in mammals have shown disparate geographical patterns for different host taxa and parasite groups across spatial and ecological scales of analysis (see review by Preisser, 2019). For instance, Guilhaumon *et al*. (2012) found no correlation between the latitude and species richness of fleas infecting mammals at a global scale, while Krasnov *et al*. (2004) found that species diversity of fleas infecting rodents decreased towards the tropics. Regarding helminth parasites, Preisser (2019) found that for cricetid rodents worldwide, species richness of nematodes in host populations was higher towards tropical areas, while the reverse was true for cestodes, and trematode diversity showed no relationship with latitude. When total species richness for the 3 helminth groups was analysed, the author found a negative relationship with latitude. Furthermore, Preisser *et al*. (2022) focusing on Central and North America cricetid rodent assemblages found similar trends for overall helminth and nematode diversity, with further variation between the ecological scales of analysis (infracommunity *vs* component community). In contrast, Harris and Dunn (2010) found that diversity of parasites (including viruses, bacteria, protozoa, helminths and ectoparasites) infecting Nearctic carnivorous mammals had a mid-latitude peak. Overall, these patterns seem to be related to or depend on ecological scale, and specific host and parasite groups, although the data sources and analyses employed to arrive at these results are also diverse and might have an influence on the results.

The demonstrated variation with latitude in species richness of animal and plant groups does not appear to be a result of latitude *per se*, but instead is most likely the result of the correlation between latitude and environmental and historical factors that affect biodiversity. Climate has been shown to be one of the most important of these factors through its influence on primary productivity, and *via* the imposition of physiological limits on species distributions and geographical range sizes, especially through seasonal variations (Willig *et al*., 2003). For parasitic organisms, another key factor that varies with latitude is the diversity of host species. According to the habitat heterogeneity hypothesis applied to parasitic organisms, parasite diversity should be positively correlated with host diversity (Johnson *et al*., 2016). Another hypothesis about the parasite–host diversity relationship put forward by Janzen (1981) states that an inverse relation between species richness and average population density in an assemblage of hosts creates a trade-off between resource diversity and scarcity, resulting in a parasite species richness peak in areas with intermediate values of host diversity. For parasites, a better recognition of biogeographical patterns is required to understand the diversity drivers and their variation among host and parasite taxa.

Most assessments of large-scale spatial variation of parasite richness have been based on reviews of available literature databases such as the Global Mammal Parasite Database (Stephens *et al*., 2017) or the London Natural History Museum Host–Parasite database (Dallas, 2016) – although see Preisser *et al*. (2022) and Johnson and Haas (2021) for assessment based on data collected in the field specifically for this purpose. These databases provide valuable spatial/geographic information of host–parasite associations reported in thousands of papers, book chapters and other sources. Nonetheless, the use of these databases has limitations for exploring geographical variation of parasite species richness, such as the coarse georeferencing of certain records (e.g. to the country level), the fact that many parasite records do not make it to the literature, and the difficulty involved in quantifying sampling effort variation from publications where the number of host specimens examined is not reported. Another problem is the fact that in many cases, publications reporting host–parasite records are not associated with voucher specimens of either host (nor parasite!), so that corroboration and verification of both host and parasite species identifications now or in the future is impossible (Hoberg *et al*., 2022*a*).

An alternative source of information for exploring large-scale parasite diversity patterns, and one that can significantly complement our current knowledge on the subject, is museum collections, where each specimen that is collected in the field at a specific and defined geographic locality and date of collection is deposited as a voucher in a permanent specimen collection in a museum bio-repository that is designed to house parasite specimens and their data for perpetuity. Specimen-based databases provide largely untapped data sources that can be used to explore the subject of parasite/host geographic distributions and overcome some of the above-mentioned limitations. The availability of voucher specimens enables taxonomic corroboration, and species identification *via* the self-correcting scientific process provided by a scientific name that, combined with more collection of specimens, can provide rapid assessments of the effect of taxonomic mistakes on the patterns that are detected. Equally important, the nature of these records allows more accurate estimation of sampling effort across space and through time, enabling researchers to account for sampling disparities when exploring diversity patterns. Despite this, collection databases are dispersed, and have their own challenges, such as non-standard georeferencing and non-standardization of host and parasite taxonomic information; however, as more specimen bioarchive databases come online, these problems will also resolve as new more powerful methods are employed to name what is found in nature (Hoberg *et al*., 2022*a*).

In this paper, we use a compiled, standardized and georeferenced database of parasite museum records to explore diversity patterns of supracommunities of helminth parasites infecting mammals over the Nearctic realm. For this, we analysed parasite occurrence data from different ecoregions using species accumulation curves to estimate species richness while accounting for the highly uneven sampling across geographical space. In this paper, our objectives are to use parasitology collections data to: (i) describe the distribution of parasite sampling effort and completeness through the study area; (ii) estimate parasite species richness over the ecoregions of the study area and its variation with latitude; (iii) assess correlations among parasite species richness and ecoregion area, ambient temperature and its variability, precipitation and its variability, and host species richness and (iv) explore these patterns at a lower host taxonomic scale using as markers, parasites from carnivores and rodents.

## Materials and methods

This work focuses on the parasite species infecting mammal assemblages at the ecological scale of the supracommunity. Here, we define a parasite supracommunity as all populations of helminth parasites which during at least one of their developmental, or life stages, infect mammalian species present within a particular ecoregion (Bush *et al.*, 1997).

**Figure 1.**
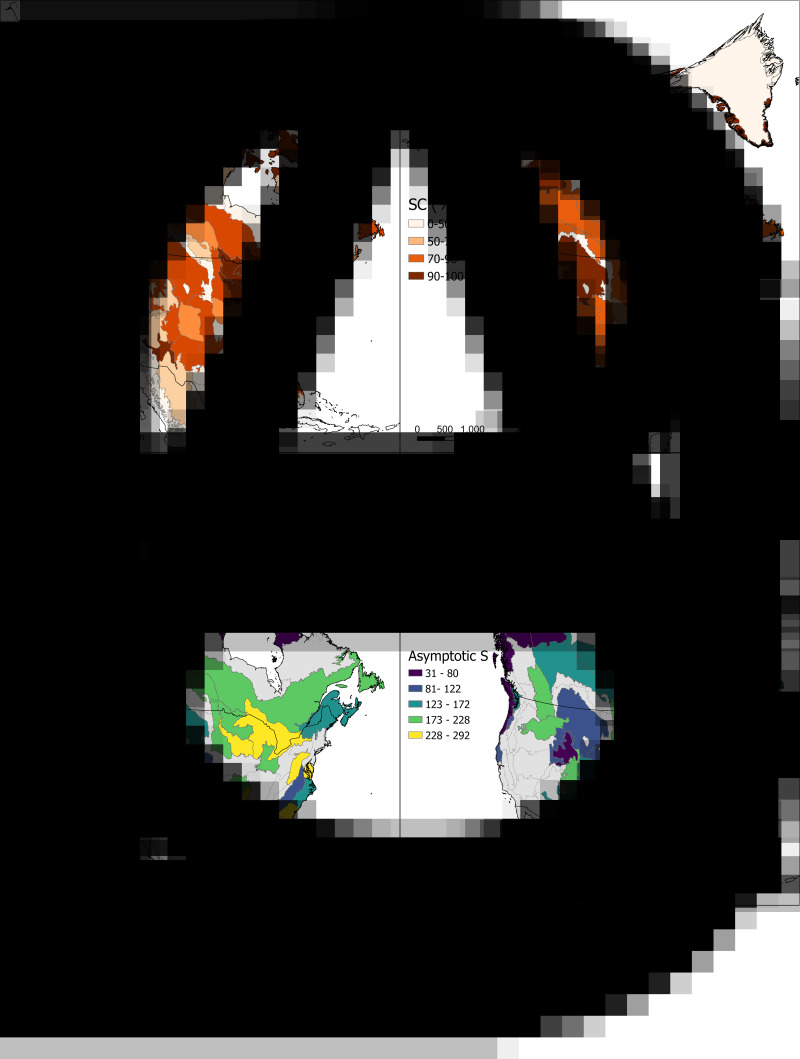
Sampling and species richness estimates for helminth compound parasite communities of mammal assemblages through Nearctic ecoregions. (A) Number of parasite specimens collected from mammals in each ecoregion. (B) Estimated SC percentage achieved by the collected specimens in each ecoregion. (C) Estimated richness at a SC of 90% for each ecoregion included in the analyses. (D) Nonparametric asymptotic estimate of total number of species for each ecoregion included in the analyses.

We selected our study area based on the definition of the Nearctic realm by the Ecoregions GIS layer of the Nature Conservancy available at (https://geospatial.tnc.org/datasets/b1636d640ede4d6ca8f5e369f2dc368b/about), and added Tropical Florida ecoregion given its continuity with the North American landmass (Fig. 1; Supplementary Fig. 1). To describe the spatial patterns of parasite supracommunity diversity in mammalian assemblages we harvested records of museum specimens from accessible parasitological collections containing the largest samples of parasite specimens from the region. Given the highly uneven sampling of parasites through space, we estimated parasite species richness through interpolation, and extrapolation of the species accumulation rates (see below). Subsequently, the correlation between estimated parasite species richness and latitude was assessed, as well as between parasite species richness and various environmental factors. This was done for supracommunities of helminth parasites from all mammalian host species, and helminths from carnivores and rodents separately. Additionally, we report the sampling effort in terms of specimens, or lots collected, from each ecoregion and sample completeness of each ecoregion.

### Parasite occurrence data

Here we exploited what we consider underutilized, open-source databases consisting of verified host and parasite location data derived from 4 different research museum collections in North America: (i) The Harold W. Manter Laboratory of Parasitology Parasite Collection; (ii) the former United States National Parasitology Collection now housed at the Smithsonian Institution; (iii) the parasite collection in the Museum of Southwestern Biology and (iv) the Parasite Collections of the Canadian Museum of Nature. In this study, the unit of analysis comprises each record in a collection database of a parasite specimen or lot of specimens coming from an individual host (herein referred to as *specimen*). Given that the number of specimens in a lot is not reported in many cases, we interpret each record from the parasitological collections as an event of detecting a parasite species from a host individual. Analysis of geographic occurrence and host/parasite association data from these databases has several important advantages; the first being the fact that actual voucher specimens in accessible museum collections are linked to the data in the database and thus provide verifiability, replicability and the opportunity to evaluate taxonomic accuracy. Thus, data coming from vouchered and identified specimens allow researchers to explicitly examine the effects of host and parasite taxonomic identification accuracy on the patterns found in various types of analyses. Additionally, data derived from museum specimens, or lots permit more precise estimations of sampling effort and include huge numbers of collection events that have never before been used in the published literature stream.

To accumulate this information for our analyses, we concatenated and standardized parasite data from the abovementioned parasitological collections. To do this, we first standardized the host taxonomic identity information to account for taxonomic changes, as well as typographical errors. This standardization was performed in R (version 4.0.1) using the package Taxize (Chamberlain *et al*., 2020). Further, we selected records from helminth parasites (Acanthocephala, Cestoda, Trematoda, Nemata) collected from species of terrestrial mammals native to the Nearctic region (Kays and Wilson, 2009; Ramírez-Pulido *et al*., 2014). Afterwards, we selected those records with geo-spatial information, that is, those records that had verified good georeferenced data or those records with a description of the locality. Then, we georeferenced those records lacking coordinate data using the locality description and the *geolocate* function from the package ggmap (Kahle and Wickham, 2013), which uses the Google maps georeferencing service. From a sample of 200 localities, we found that the georeferencing was accurate to within a 50 km radius of accuracy for 99% of the records. Finally, we standardized parasite taxonomy and included only records with taxonomic identification to species or genus in the final database.

One of the main problems we found with museum database records was that a significant number of specimens in the parasite collections are derived from experimental infections, which have no value for our question. Three steps were taken to remove experimental or stamp collecting types of records from the databases. First, records tagged as experimental infections with parasites were removed. After this, records coming from zoos, veterinary hospitals and research laboratories were removed. Finally, we eliminated those records of parasites coming from specific localities that included records from hosts that were non-native species and/or experimental infections.

### Parasite richness estimates

For each ecoregion, we extracted the parasite collection records coming from within its boundaries. Within each ecoregion, we only included specimens without species identification (i.e. assigned only to genus) when none of specimens identified to species corresponded to that genus. This was done in order to reduce the chances of counting the same taxon twice and bias our estimates. Afterwards, we employed species accumulation curves, using collected specimens (or specimen lots) as a replicate sample to compare species richness among ecoregions. We estimated species richness using 2 different approaches, 1 is rarefaction comparisons, and the other is total richness estimates.

In the first approach, we estimated species richness found at a sample coverage (SC) of 90% (hereafter, called the rarefied estimate). Here, SC is defined as the proportion of the total number of individuals in a community that belongs to the species represented by the actual specimens in the collection. SC has been suggested to be a preferable way of comparing diversity between and among communities rather than measures of sampling effort given that SC is less sensitive to differences in species abundance distributions than rarefaction based on sampling effort (Chao and Jost, 2012). We used the Chao and Jost (2012) coverage-based rarefaction and extrapolation method to interpolate or rarefy the richness estimate for ecoregions with an SC above 90%, and extrapolated the expected richness for ecoregions with lower SC values. This approach provides comparable richness estimates among all ecoregions independent of sampling efforts or large differences in the probabilities of species making it to the sample due to variation in prevalence and abundance. For carnivores and rodents, we also compared species richness among ecoregions using the rarefied estimate to 90% of SC.

Our second approach to explore parasite diversity was to estimate the total species richness for each ecoregion using the non-parametric estimator proposed by Chao *et al*. (2014), which uses the number of species with only 1 and 2 records to infer the number of non-detected species (hereafter, called the asymptotic estimate).

The sampling completeness, rarefied/extrapolated and asymptotic species richness estimates for each ecoregion were calculated using the R package iNEXT (Hsieh *et al*., 2016). For the analysis including all mammalian hosts, we report the rarefied and asymptotic richness estimates. For subsets of carnivores and rodents, we only report the rarefaction estimate given that for small samples extrapolation can introduce bias in the estimators (Chao *et al*., 2014).

We also explored the level of representation of host assemblages in the collections for each ecoregion by estimating the Sørensen dissimilarity index between the mammal genera sampled, and the genera with predicted distributions within each ecoregion (see below) using the R package vegan (Oksanen *et al*., 2022). In this paper, we only report parasite species richness and its relationship with latitude and environmental factors for ecoregions having an SC higher than 70%, and a maximum Sørensen dissimilarity index value of 0.6 between the sampled and potentially present host genera. This sampling threshold was selected as a compromise between having an accurate representation of parasite diversity through the study area and having an adequate parasite and host sample of each ecoregion.

### Latitude and environmental correlates of parasite species richness

As our parasite richness estimates include associated uncertainty (Chao and Jost, 2012), we used a Bayesian inference framework to evaluate the relation between latitude and parasite diversity while accounting for estimation error. To do this, we modelled the relation between latitude and the unknown real species richness of each ecoregion. Each observation then is drawn from a normal distribution centred at the real species richness value with the standard deviation provided by each richness estimate as shown below:

- *S*_est,*i*_ ~ Normal(*S*_true,*i*_, *S*_SE,*i*_)
- *S*_true,*i*_  ~ Normal(*μi*, *σ*)
- *μi* = *α* + *β*Lat × *Latitude_i_*
- *α* ~ Normal(0, 3)
- *β*Lat ~ Normal(0, 1)
- *σ* ~ Exponential(1)

where *S*_est_ is the richness estimate (rarefaction or asymptotic), *S*_SE_ is the estimated standard error of the estimate, *S*_true_ is the true, unknown richness at each ecoregion and *β*Lat is the rate of change in diversity with latitude. As some parasite groups have been shown to display mid-latitude peaks in diversity (Janzen, 1981; Harris and Dunn, 2010; Preisser *et al*., 2022), we also evaluated the fit of a quadratic relation between parasite richness and latitude.

To examine and explore the correlation of parasite richness with some biotic and abiotic variables previously suggested by Willig *et al*. (2003), Guégan *et al*. (2005) and Preisser (2019) to be important factors influencing free-living or parasitic biodiversity patterns, we used the same Bayesian approach noted above. For this part of the analysis, the abiotic variables include: ecoregion area, mean annual temperature, temperature seasonality (standard deviation), annual precipitation and precipitation seasonality (variation coefficient). We obtained these climatic data from the WorldClim database (Fick and Hijmans, 2017) at a resolution of 1 km^2^, and then averaged the pixel values across each ecoregion. Besides, we estimated mammal species richness within each ecoregion as the number of species whose range overlapped the ecoregion base on the IUCN red list assessment geographic range estimations (IUCN, 2016). The response and predictor variables were standardized by subtracting the mean and dividing by the standard deviation.

The models were fitted using software *Stan* through the function *ulam* of the R Rethinking package using Markov chain Monte Carlo estimations to draw 8000 samples from 4 chains (McElreath, 2020; Stan Development Team, 2022). Chain convergence was evaluated using the Gelman–Rubin convergence diagnostic ‘R-hat’ for each parameter, from which values lower than 1.01 were taken as an indication of convergence towards the estimate. Additionally, rank plots were checked to ensure the models performed a non-biased exploration of the parameter values. We report the average posterior value and a 95% credibility interval for the model parameters as evidence for the influence of each variable on parasite species richness. We also report the proportion of the variation in estimated parasite diversity explained by each univariate model (*R*^2^).

## Results

### Sampling and species richness variation among ecoregions

The compiled-georeferenced database of helminth parasite specimens collected from mammals in the Nearctic comprises 25 131 records. Parasite sampling from wild-caught hosts is extremely uneven among ecoregions, with the number of records ranging from 0 to 2698 (average = 177; Fig. 1A). Thirty-six out of 103 ecoregions were not represented in the parasitological collections compiled by us, and will not be discussed further here. The number of records per ecoregion is not correlated with latitude (Supplementary Fig. 1). Nevertheless, the similarity between the collected and potential host assemblages, as well as sampling coverage showed a significant positive correlation with latitude (Supplementary Figs 2 and 3). This was mainly the result of less diverse actual or potential host communities towards higher latitudes. Estimated SC varied between 24 and 100%, with an average of 78% (Fig. 1B). For analyses of diversity patterns of the parasite supracommunity, we used 45 ecoregions that have an estimated sampling completeness above 70%, and a maximum Sørensen dissimilarity index value between sample and potential host assemblage of 0.6. Observed diversity by ecoregion varied from 23 species in the southern Arctic, to 177 species in the Great Lakes ecoregion (mean = 75). Rarefied richness estimates ranged between 12 and 161 species in the Bering Sea and Aleutian Islands and the Great Lakes ecoregions respectively (mean = 90; Fig. 1C). Total estimated parasite diversity (asymptotic) varied between 31 and 292 species in the Boreal Cordillera ecoregion (in northern British Columbia), and the Great Lakes ecoregion, respectively (mean = 139; Fig. 1D). Results for each ecoregion are presented in Supplementary material S2.

**Figure 2.**
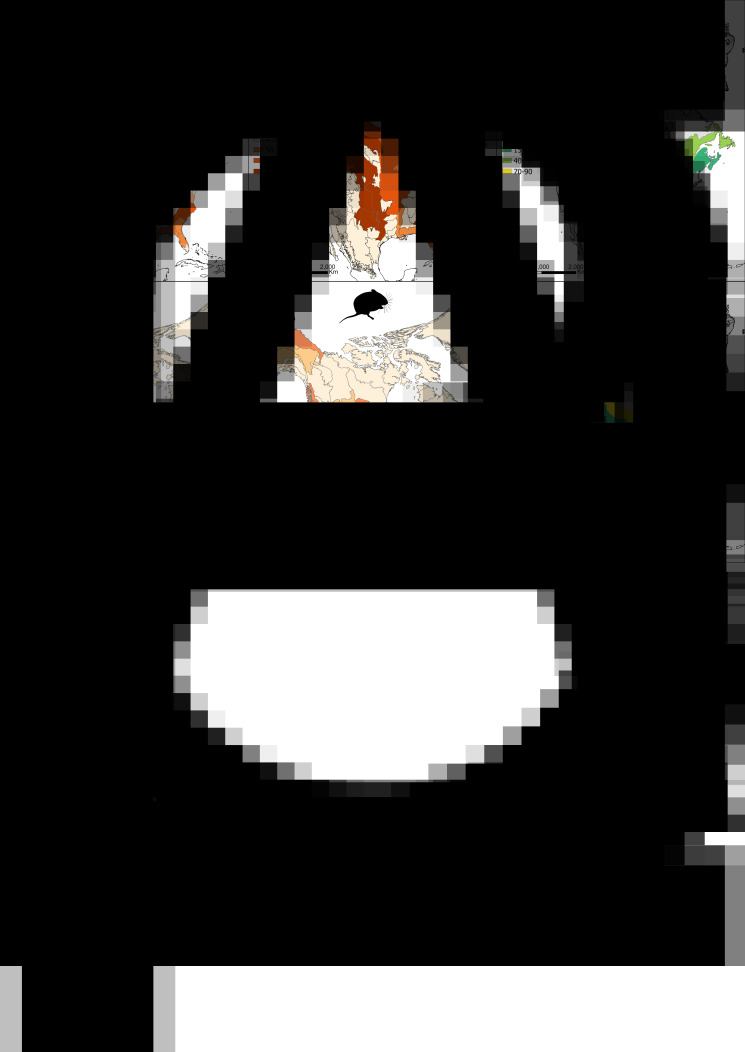
Number of parasite specimens collected from each ecoregion, estimated SC percentage for parasites achieved by the collected specimens in each ecoregion and estimated richness of parasites at an SC of 90% for parasites infecting carnivores (A–C) and rodents (D–F).

For carnivores, we extracted 9586 specimen records from all verified databases, ranging from 16 to 1669 for each ecoregion (Fig. 2A). We selected 32 ecoregions meeting the sample completeness and host representation thresholds. Carnivore sampling coverage of these areas ranged between 74 and 100% (average = 87%; Fig. 2B). The observed helminth parasite diversity in carnivores varied from 8 species in the Boreal Plain, to 60 species in the Mid-Atlantic Coastal Plain (mean = 27, Fig. 2B). Rarefied estimates ranged between 6 and 90 species in the temperate coniferous forests and the Prairie–Forest Border, respectively (mean = 90). The results by ecoregion for carnivore assemblages are presented in Supplementary material S3.

Rodents have 8230 verifiable records, ranging from 11 to 2670 per ecoregion (Fig. 2D). Twenty-three ecoregions met the thresholds to be included in the analysis. Rodent parasite fauna sampling completeness in the included ecoregions was estimated to range between 72 and 99% (average = 84%; Fig. 2E). The observed helminth parasite diversity in rodents varied from 14 species in the Piedmont ecoregion of eastern North America, to 72 species in the Great Lakes ecoregion (mean = 30). The rarefaction estimate varied from 11 species in the Piedemont ecoregion, to 73 in the East Gulf Coastal Plain ecoregion (mean = 36, Fig. 2F). The results by ecoregion for rodent assemblages are given in Supplementary material S4.

### Latitudinal patterns in parasite species diversity

**Figure 3.**
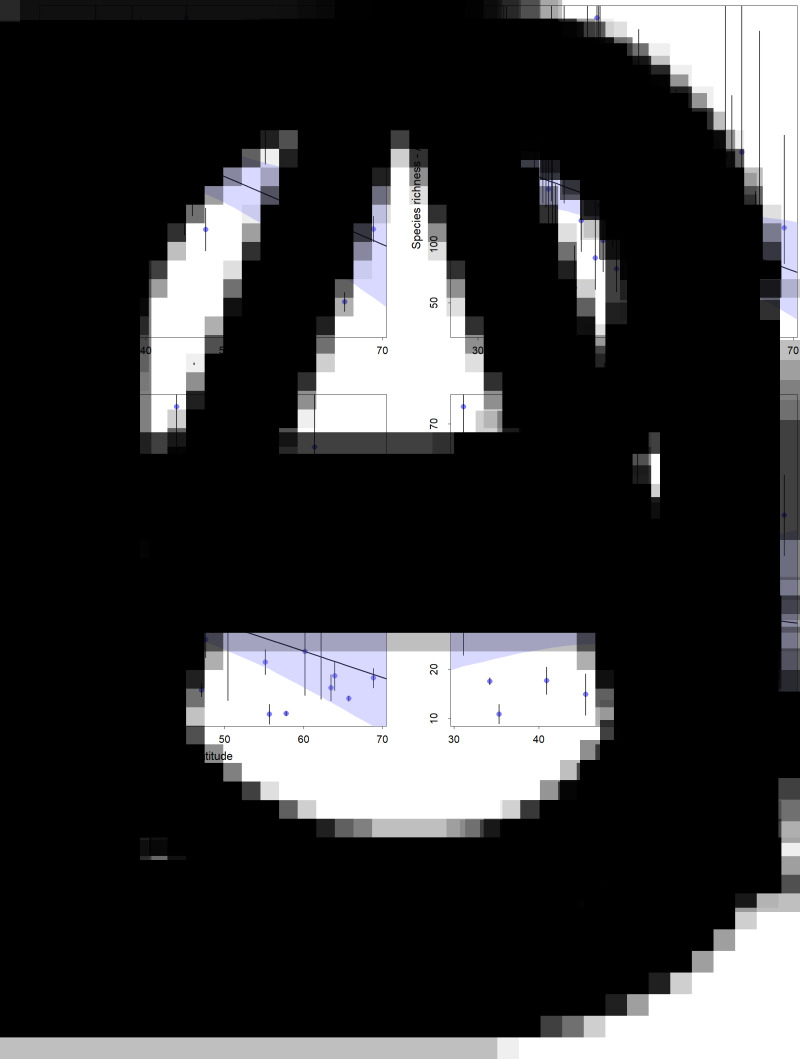
Scatterplot and fitted regression model between latitude and richness estimates for helminth compound community. (A) Estimated richness for parasites in entire mammal assemblages at an SC of 90% for each ecoregion included in the analyses. (B) Nonparametric asymptotic estimate of total number of species of entire mammal assemblages for each ecoregion included in the analyses. (C) Estimated richness for parasites infecting carnivore assemblages at an SC of 90%. (D) Estimated richness for parasites infecting rodent assemblages at an SC of 90%. Upper and lower confidence intervals for the diversity estimates are included as bars for each point. The 95% credibility intervals for the regression models are presented as shaded regions.

With visual inspection, all parameter estimates presented well mixing of the chains, suggesting adequate exploration of the parameters and 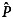 values for all parameters were 1.0. We found a negative linear correlation between parasite supracommunity richness in mammals and latitude using both the rarefaction and the asymptotic estimates of diversity (Fig. 3; Table 1). The percentage of variation explained by latitude was 22 and 18% for the rarefaction and asymptotic estimates, respectively. The quadratic models displayed similar explained variation for the rarefied and asymptotic estimates (24 and 19%, respectively), but only the first term of both models appeared to be different from 0, suggesting a decay in richness from the lower latitudes instead of a peak (Supplementary Figs 3 and 4).

**Table 1.** Results of univariate regression models exploring the relation between parasite supracommunity species richness and abiotic and biotic variables

Model variable	Posterior mean of coefficient	s.d.	2.5%	97.5%	n_eff	Rhat4	*R*^2^
Rarefied estimate – all mammals							
Lat	−0.46	0.14	−0.74	−0.19	6313	1.00	0.22
LatQ-1	−0.40	0.15	−0.68	−0.10	2379	1.00	0.24
LatQ-2	−0.15	0.15	−0.44	0.16	2131	1.00	–
Hosts	0.09	0.14	−0.20	0.37	3595	1.00	0.01
HostQ-1	0.29	0.15	−0.01	0.59	3142	1.00	0.22
HostQ-2	−0.36	0.11	−0.59	−0.15	2252	1.00	–
Temperature	0.45	0.14	0.18	0.71	4243	1.00	0.21
Temp season	0.09	0.15	−0.22	0.39	3754	1.00	0.01
Precipitation	−0.09	0.16	−0.39	0.22	3836	1.00	0.01
Prec season	−0.38	0.15	−0.67	−0.10	4157	1.00	0.15
Area	−0.01	0.15	−0.31	0.30	4220	1.00	0.00
Asymptotic estimate – all mammals							
Lat	−0.46	0.14	−0.72	−0.17	3418	1.00	0.18
LatQ-1	−0.42	0.15	−0.71	−0.12	2688	1.00	0.19
LatQ-2	−0.08	0.15	−0.37	0.20	2019	1.00	–
Hosts	0.15	0.16	−0.15	0.46	3661	1.00	0.02
HostQ-1	0.27	0.17	−0.06	0.60	3373	1.00	0.10
HostQ-2	−0.22	0.12	−0.46	0.01	2603	1.00	–
Temperature	0.40	0.14	0.13	0.67	3321	1.00	0.15
Temp season	−0.02	0.15	−0.30	0.28	3715	1.00	0.00
Precipitation	−0.02	0.15	−0.33	0.29	4332	1.00	0.00
Prec season	−0.35	0.15	−0.64	−0.06	3484	1.00	0.13
Area	−0.05	0.15	−0.36	0.23	4918	1.00	0.00
Rarefied estimate – carnivores							
Lat	−0.37	0.15	−0.65	−0.07	3220	1.00	0.09
LatQ-1	−0.37	0.17	−0.70	−0.03	3715	1.00	0.10
LatQ-2	−0.05	0.16	−0.36	0.25	2629	1.00	–
Hosts	−0.05	0.17	−0.41	0.27	3659	1.00	0.01
HostQ	−0.09	0.18	−0.44	0.27	2960	1.00	0.05
HostQ	−0.14	0.13	−0.39	0.11	2512	1.00	–
Temperature	0.34	0.16	0.04	0.66	3030	1.00	0.09
Temp season	0.12	0.17	−0.22	0.45	3723	1.00	0.01
Precipitation	−0.08	0.17	−0.41	0.28	3247	1.00	0.01
Prec season	−0.26	0.15	−0.56	0.05	2870	1.00	0.07
Area	0.01	0.17	−0.30	0.34	3782	1.00	0.00
Rarefied estimate – rodents							
Lat	−0.13	0.22	−0.58	0.29	2017	1.00	0.04
LatQ-1	−0.12	0.23	−0.56	0.34	3468	1.00	0.03
LatQ-2	−0.01	0.30	−0.60	0.59	1758	1.00	–
Hosts	0.09	0.21	−0.32	0.50	2606	1.00	0.00
HostQ	0.34	0.29	−0.26	0.93	1917	1.00	0.07
HostQ	−0.18	0.15	−0.46	0.11	1777	1.00	–
Temperature	0.02	0.23	−0.43	0.46	2479	1.00	0.00
Temp season	0.04	0.24	−0.41	0.52	3001	1.00	0.01
Precipitation	0.06	0.22	−0.37	0.49	2899	1.00	0.00
Prec season	−0.14	0.22	−0.58	0.29	3326	1.00	0.04
Area	0.23	0.21	−0.20	0.64	3243	1.00	0.04

The biotic variable models include the linear (hosts) and quadratic relations (HostQ-1 and HostQ-2) of mammals species richness at each ecoregion with parasite supracommunity species richness. The abiotic variables include the linear (Lat) and quadratic relations (LatQ-1 and LatQ-2) of latitude with parasite supracommunity species richness, as well as the linear effects of average annual temperature, temperature seasonality, annual precipitation, precipitation seasonality and ecoregion area.

For carnivores, we also found evidence of higher diversity at lower latitudes, with the posterior *β* coefficient of latitude at −0.37 (CI: −0.65 to −0.07) and latitude explaining 9% of the variation in parasite diversity (Fig. 3; Table 1). Interestingly, the diversity of helminths parasitizing rodents had no correlation with the latitude of ecoregion (Fig. 3; Table 1).

### Biotic and abiotic correlates of parasite species diversity

**Figure 4.**
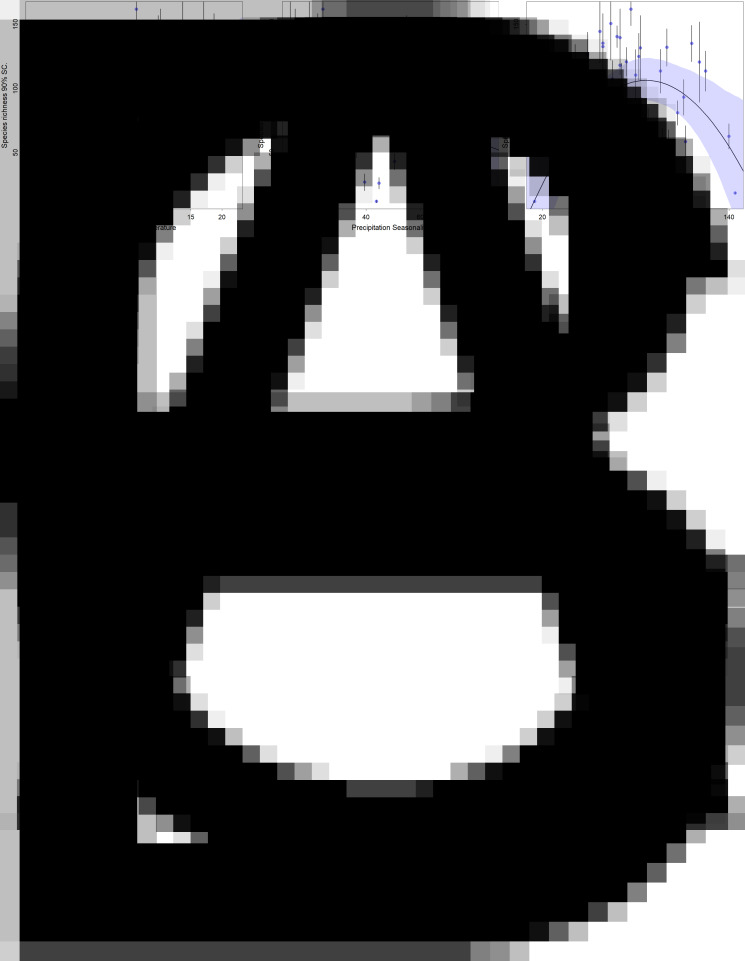
Correlation between richness of helminth compound communities in mammals at an SC of 90% for each ecoregion and host species richness (A), mean annual temperature (A), precipitation seasonality (B) and host species richness (C). The 95% credibility intervals for the regression models are presented as shaded regions.

For parasite communities in mammal assemblages, we found that the estimate of the rarefied richness is positively correlated with mean annual temperature (*β* = 0.45, CI: 0.18–0.71), and negatively correlated with precipitation seasonality (*β* = −0.38, CI: −0.1 to −0.67; Fig. 4; Table 1). Area, temperature seasonality, precipitation and host species richness do not show a significant linear correlation with this estimate of parasite species richness. After visual inspection of the data, we decided to fit a quadratic model to the host species richness variable, which resulted in significant coefficients and explained 21% of the variation in parasite species richness (Fig. 4; Table 1). Using the asymptotic estimated richness we found the same significant correlations for the 2 abiotic variables, but not for host species richness (Table 1).

For parasites infecting carnivores, we found that mean annual temperature and precipitation seasonality present a positive and negative correlation, respectively, with the parasite rarefaction estimate of diversity. Interestingly and perhaps not surprisingly (see ‘Discussion’), rodent parasite faunal diversity was not correlated with any of the variables explored in this study.

## Discussion

To our knowledge, this is the first study utilizing the huge potential of museum databases of parasite collections from wild-caught animals for exploring macroecological and biogeographical patterns of parasite diversity at large geographical scales. The dataset we used enabled us to use species accumulation curves to study parasite diversity patterns over space, and compare richness estimates under the acute and pervasive problem of uneven and incomplete sampling. Also, this is one of the few studies of large-scale parasite diversity gradients where species richness is estimated for specific regions (but see Dallas *et al*., 2018), instead of species richness being estimated for species or populations of hosts – i.e. parasite supracommunity. With this novel dataset and analysis approach for parasites, we found a clear latitudinal diversity gradient in the helminth parasite supracommunity of mammalian hosts across the Nearctic region.

The latitudinal gradient of higher helminth species richness in mammals towards lower latitudes has been mentioned by other authors working at different scales and with different data sources. Preisser (2019) found higher species richness towards the tropics in helminth communities of cricetid rodents (although the pattern was most likely driven by the diversity gradient of the Nemata). Besides, Guernier *et al*. (2004) found a latitudinal gradient for human parasites and Dallas *et al*. (2018) found a latitudinal gradient in helminths of vertebrate hosts. On the contrary, Bordes *et al*. (2010) did not find a correlation between parasite richness and latitude for helminth parasites of mammalian species at a global, host species/population scale. Similarly, Nunn *et al*. (2005) found a diversity gradient for protozoan parasites from primates but not for their helminth fauna. Several studies of mammalian ectoparasites have found contrasting effects of latitude. Of these studies, only Preisser *et al*. (2022) based her data on actual collections. Nevertheless, our results should be compared with some caution given that in this work we explore the diversity gradient above 27° north, missing an important part of the picture of parasite/mammal diversity from the tropical ecoregions.

We also found that latitude had a significant effect on parasite supracommunities of carnivores. This result contrasts with the mid-latitude peak in parasite species richness shown by Harris and Dunn (2010) for carnivores in the same large-scale geographic study area using a host-filling approach. Also, in contrast to our results, Lindenfors *et al*. (2007) found a positive relation between latitude and parasite species richness at a carnivore species scale.

Interestingly, for parasites from rodent hosts, no latitudinal pattern in diversity was found. This result may be due to the more subtle ways that most rodents live in the ecosystems as was speculated by Haverkost *et al*. (2010) where the estimate of parasite distributions over geographic space was clearly shown in the Great Plains and Gulf Coast biomes but no predictions could be made in the Arctic Tundra biome. These authors suggested this may be due to the subnivean habitat occupied by the rodent hosts over much of the year. Rodent associations with subterranean burrow might be sheltered to a certain degree from climatic conditions, rendering other environmental factors more important in shaping their diversity patterns. This contrasts with the results of Preisser (2019) who found a latitudinal gradient for helminths from an analysis of only rodents of the family Cricetidae. These divergent results might arise from the taxonomic scale of the analyses, with rodents showing different patterns at the family scale. Another possible explanation for the disparity between our studies is that the analysis by Preisser (2019; Preisser *et al*., 2022) was performed at more local scales, rather than the grand scale of the ecoregion, and that local diversity filters in rodents may result in a latitudinal gradient, while ecoregional filters may not show the gross result of a latitudinal gradient in diversity. This is a set of questions that needs further exploration.

An unexpected result of this work is the trend of a peak in helminth diversity in ecoregions of intermediate levels of host species richness. These results are influenced by the ecoregions with parasite diversity at the extremes of host diversity, and further collection-based data will be needed to test these in the future. To our knowledge, this is the first time that such a pattern has been documented for helminths of terrestrial vertebrates, and this contrasts with the results reported by the abovementioned authors of a linear or no correlation between hosts and parasite diversity. However, Janzen (1981) found a mid-latitude peak in the diversity of parasitoid wasps of the family Ichneumonidae and he attributed this pattern to intermediate values of host species richness at these latitudes. Leading the way as usual in cutting-edge ecological studies, Janzen (1981) suggested that the higher host species richness in the tropics results in abundance per species being so low that the host resource itself was not sufficient for species above a certain level of specialization, while at higher latitudes host species richness was too low to sustain higher diversity. Further research should evaluate the generality of the pattern found in this work, and explore potential responsible mechanisms. Janzen's hypothesis would be a good start as data on relative species abundance, as well as parasite associations are available; however, there is a significant massive dearth of data on intermediate hosts for many of the parasites that have complex life cycles. In turn, this relationship between host and parasite species can deepen our understanding of the effects of changing host assemblages over parasite supracommunities. The fact that no correlation was found between either carnivore species or rodent species richness and their parasitic faunal richness might be due to the importance of intermediate hosts for many of their parasites, and is a topic that also requires further exploration. It is interesting that for many of the carnivores that have parasites with complex life cycles, especially cestodes of the family Taeniidae, the intermediate host is a rodent or perhaps a lagomorph (Abuladze, 1964).

Besides latitude and host species richness, environmental variables also showed an effect over ecoregional helminth parasite species richness. Specifically, for parasites of mammal and carnivore assemblages, mean annual temperature and precipitation seasonality were important factors correlated with parasite diversity. Temperature has been suggested as one of the main factors driving the latitudinal gradient of free-living organisms through its effects on the probability of persistence of populations and rates of speciation (Brown, 2014); while reduced seasonality has been argued to have positive effect on diversity by accentuating the effect of geographical barriers (through reduced evolved environmental tolerances), thus promoting allopatric speciation (Ghalambor *et al*., 2006). Seasonal variation in temperature and precipitation may also influence parasite diversity in this way, but this is unclear. Alternatively (or in conjunction), several parasite-specific mechanisms might be the result of the influence of temperature and seasonality on parasite diversity. These mechanisms might include enhanced transmission through higher rates of survival of free-living stages, greater abundances or diversity of hosts and thus increasing the probability of persistence and providing chances for speciation, as well as host physiology influencing parasite transmission (Zamora-Vilchis *et al*., 2012; Møller *et al*., 2013; Gehman *et al*., 2018).

We found no relation between the number of specimens in collections and latitude, suggesting that although highly uneven, the sampling effort does not seem to be a significant bias along this gradient. On the contrary, SC did show a weak, but significant, correlation with latitude. Higher SCs were achieved for ecoregions of northern latitudes. We interpret this as the result of lower diversity of species at higher latitudes resulting in more easily sampled parasite supracommunities. We believe that this SC–latitude relation has a minimal impact on our results and their interpretation given the fact that the rarefied richness estimates come from the interpolation or extrapolation to a defined SC value and those data coming from low SC regions were not included in our analyses.

Despite our data-curation approach, utilizing and analysing large-scale data derived from completely databased biological collections, there are some shortcomings of our analysis. First is that species accumulation curves based on individuals assume a random sample of individuals being taken from a biological community. For many of the samples stored in parasite collections we analysed, this may depart significantly from a random collection, as specimens deposited in each bioarchive depend on the level of effort by individual scientists to trap target hosts and parasite groups. For example, some individual collectors or even whole collecting expeditions focus on fleas and cestodes and sample the nematode and trematode fauna in an incomplete way, throwing out hundreds of individual parasites and saving only 10 ‘representative individuals from each host encountered’. Nonetheless, we assume here that by including data and associated specimens over a broad temporal frame at large spatial scales will make up for the lack of random collecting events, because each ecoregion has samples coming from different collecting expeditions or different collectors with different target host mammal groups collected at different times. Furthermore, by focusing our analyses on ecoregions for which there is a host sample at least partially resembling the regional species pool (maximum Sørensen dissimilarity index of 0.6), we believe we have a representative set of specimens for each of the ecoregions included in the analysis. This difference between sampled and potential hosts did show a correlation with latitude, stemming from the fact that host communities are simpler at higher latitudes. Nevertheless, this does not affect our conclusion because we selected only the ecoregions with a representative host sample, and because this issue would tend to inflate diversity estimates at higher latitudes, the opposite of our findings. A potential source of bias in our analysis is identification of species or changes in the taxonomy of species names through time, and it is one that should be further explored in future studies, with additional field collections and comparative work being done. However, species names of specimens collected in the field are self-correcting and work to identify specimens at the species level will continue to be refined as the science of species-level biodiversity estimates becomes more robust (Hoberg *et al*., 2022*b*). Nevertheless, as the analyses are done at an ecoregional scale, we believe there will be fewer problems with synonyms from different areas in the estimation of species richness.

Parasite community organization is hierarchical, although few community assembly rules (*sensu* Weins, 1989), besides snapshots of evidence of distributions, have yet been discovered (Moss *et al*., 2020). In general, it would not be expected that the composition of communities of parasites, that is species making up interacting groups, would have different drivers of assembly than those discovered for free-living animals such as birds (Weins, 1989). It is clear that parasite communities have a somewhat nested organization ranging from infracommunities within host individuals, through component communities in host populations, to supracommunities infecting all potential hosts (Holmes and Price, 1986; Bush *et al*., 1997; Poulin, 1998). What is not so clear is how parasites move through potential host individuals, populations and communities and how and when these parasites might switch hosts to make the community structure that we get temporally based snapshots of; although the general process is becoming more clear as was speculated by Janzen (1981) and Agosta (2006) and summarized, reviewed and shown by Agosta, Janz and Brooks (2010) and Agosta (2022).

One point that we make here is that a sound understanding of parasite species richness patterns and parasite and host community properties must take into consideration all scales. We believe that accounting for variation at all scales will not only reconcile disparate empirical results but will also provide useful information regarding the discovery of the mechanisms and processes that are producing these patterns. For example, parasite supracommunity species richness is the result of both species richness in component communities, as well as parasite species turnover among host populations. Thus, measuring both quantities can allow researchers to disentangle the drivers of richness of parasites. In this sense, our estimates of helminth supracommunity richness in mammals might represent a useful dataset over which to compare component and infracommunity richness. Also, estimations at the ecoregion level can enable analysis of the effects of regional and local processes in structuring communities as it can be a quantification of species pools available to local host populations or assemblages within each ecoregion (Weins, 1989).

## Data Availability

The concatenated specimen dataset is hosted on Lamarck at the H.W. Manter Laboratory of Parasitology web page. The R code used to estimate parasite species richness, and analyse the relation between parasite diversity and the selected covariates is displayed at the GitHub repository: https://github.com/Sebastianbotero/Parasite-compound-communities-latitudinal-diversity-gradients.

## Supplementary material

For supplementary material accompanying this paper visit https://doi.org/10.1017/S0031182023000458. click here to view supplementary material
