# Supplementary material for: Tapping into natural history collections to assess latitudinal gradients of parasite diversity
Source: Parasitology. 2023 May 9;150(8):723–33. doi: 10.1017/S0031182023000458 (PMC10410379; doi:10.1017/S0031182023000458)
Supplement: Supplementary file 1 [file S0031182023000458sup.zip › S0031182023000458sup002.pdf]

S1. Supplementary Figures

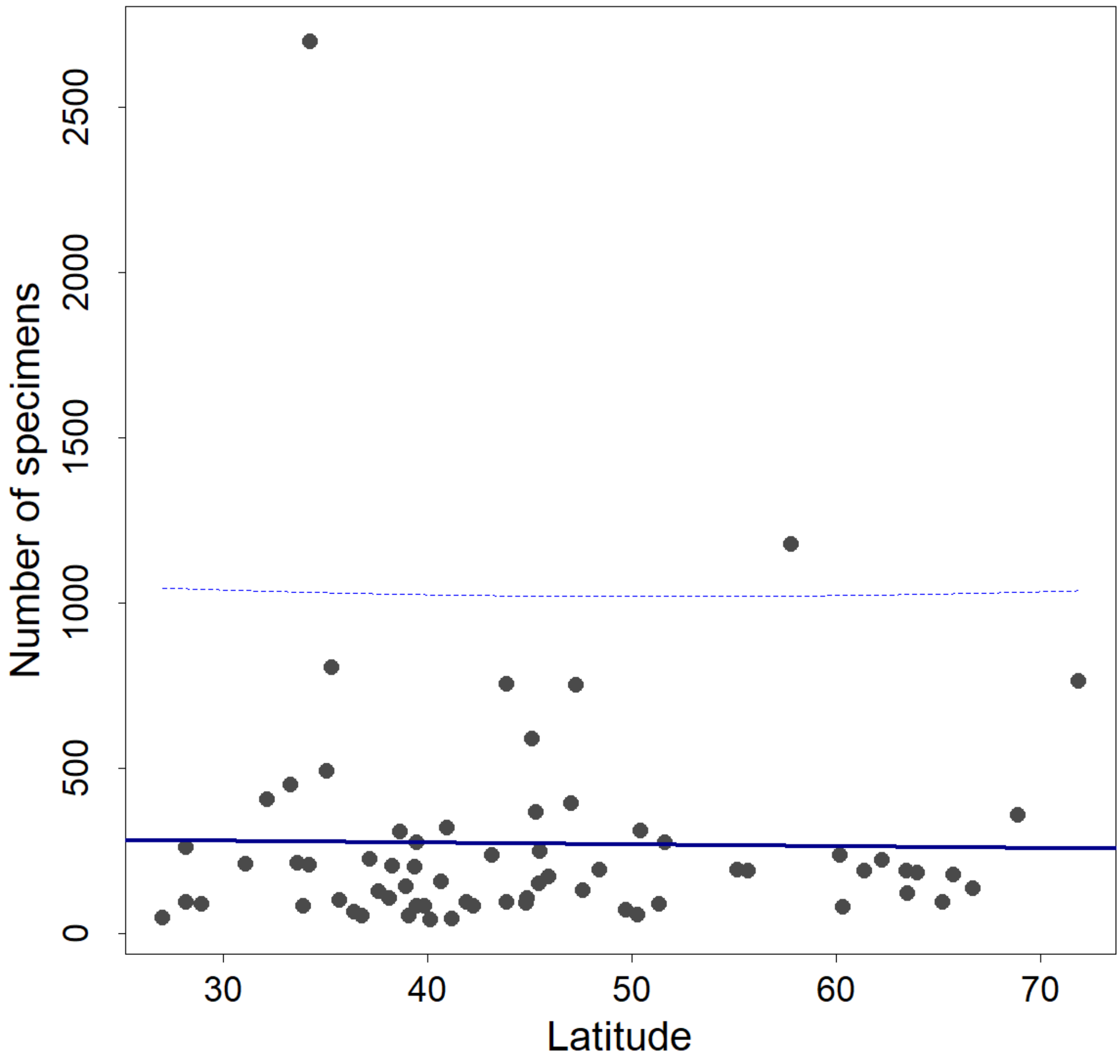

Supplementary Figure 1. Scatterplot and fitted linear regression of the relation between the latitude at the centroid and the number of parasite specimens collected within each ecoregion. There is no significant relation between sampling intensity and latitude (P=0.89).

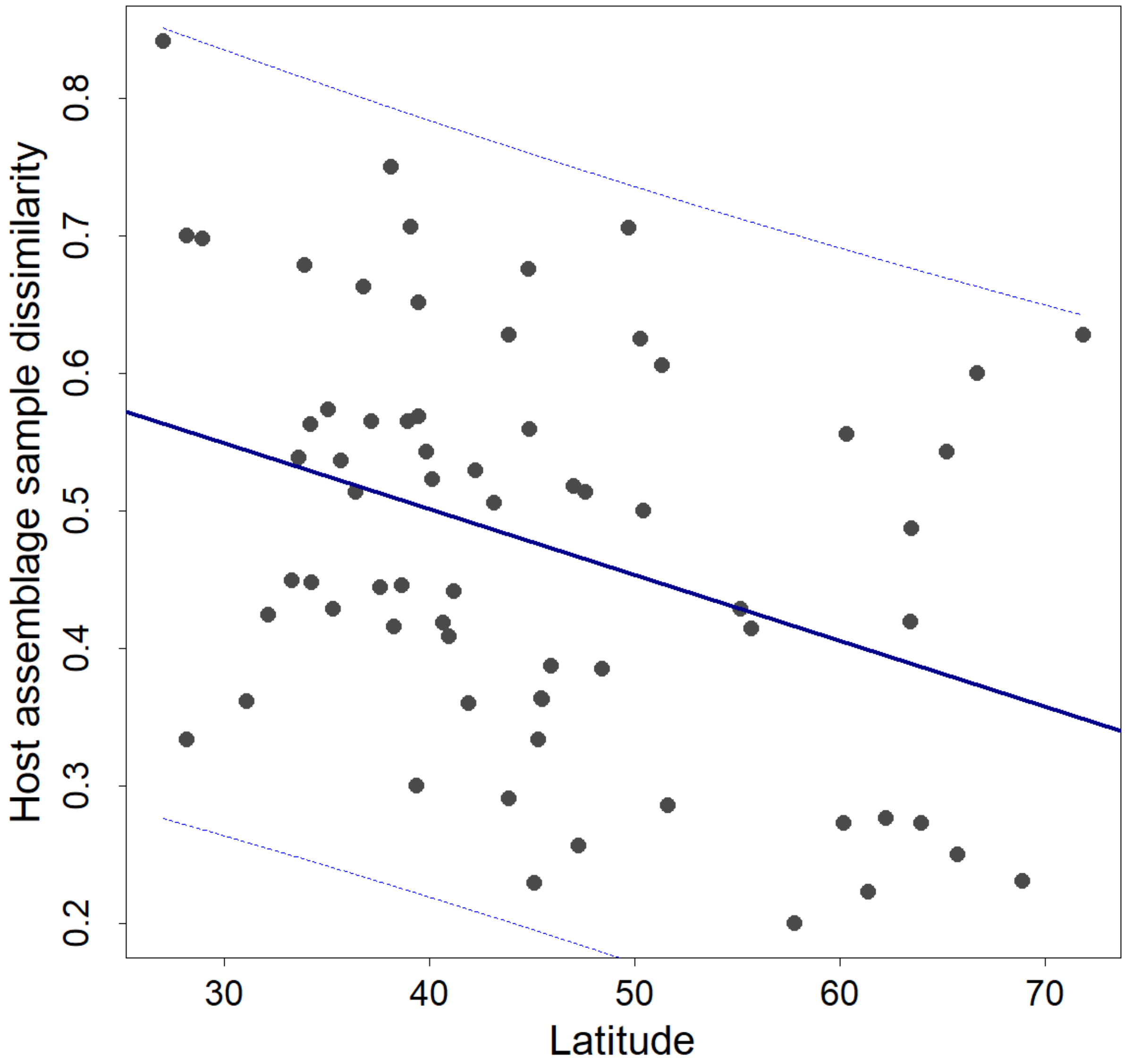

Supplementary Figure 2. Scatterplot and fitted linear regression of the relation between the latitude at the centroid and host representation level within each ecoregion. There is a significant relation between sampling host representation level and latitude (P=0.009). Host representation level is estimated as the Sorensen dissimilarity index between the genera from which parasites were collected, and the genera potentially present at each ecoregion.

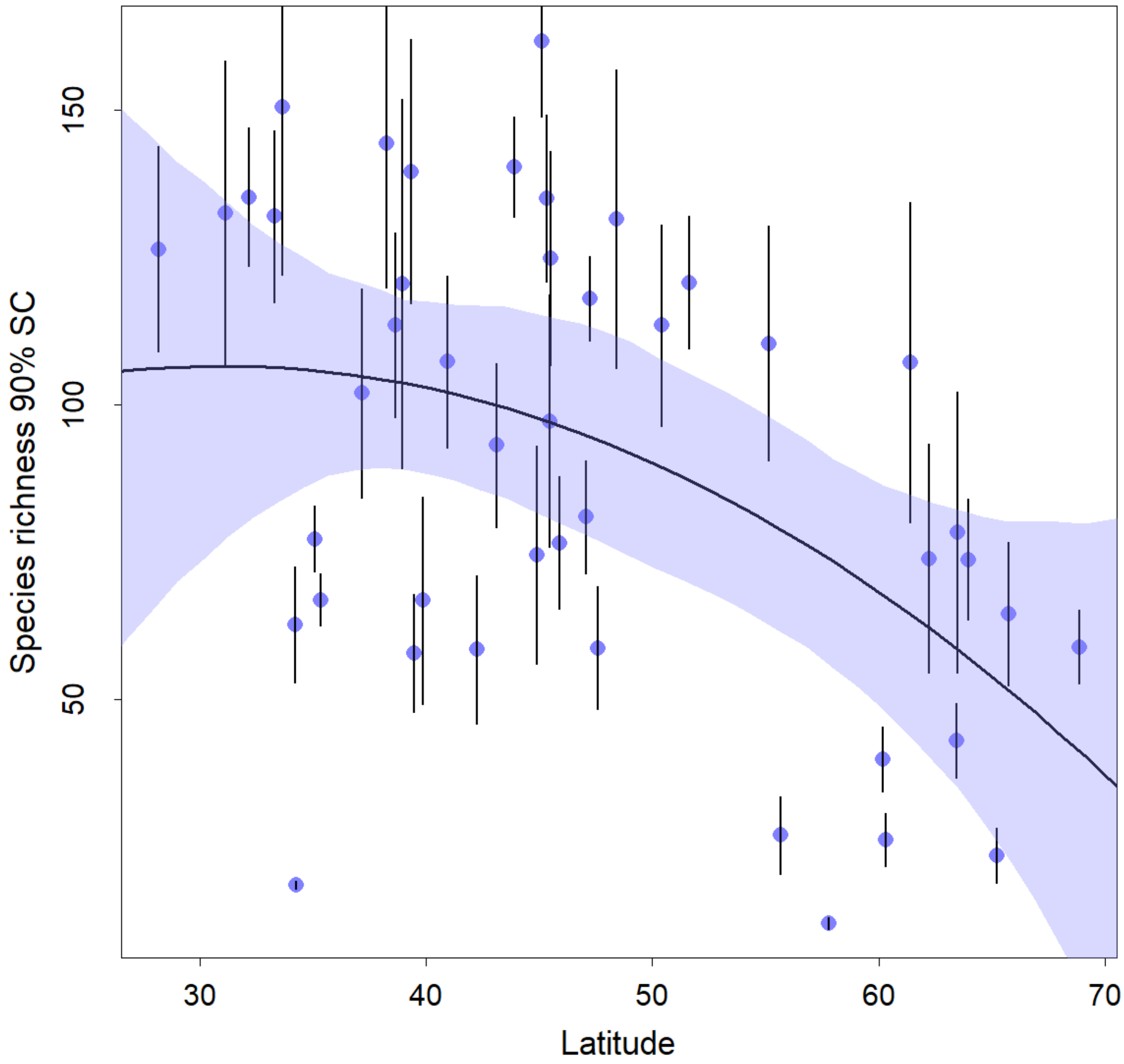

Supplementary Figure 3. Scatterplot and fitted quadratic regression of the relation between the latitude at the centroid and estimated richness for parasites in entire mammal assemblages at a sample coverage of 90% for each ecoregion included in the analysis. Upper and lower confidence intervals for the diversity estimates are included as bars for each point. 95% credibility interval for the regression models are presented as dashed lines.

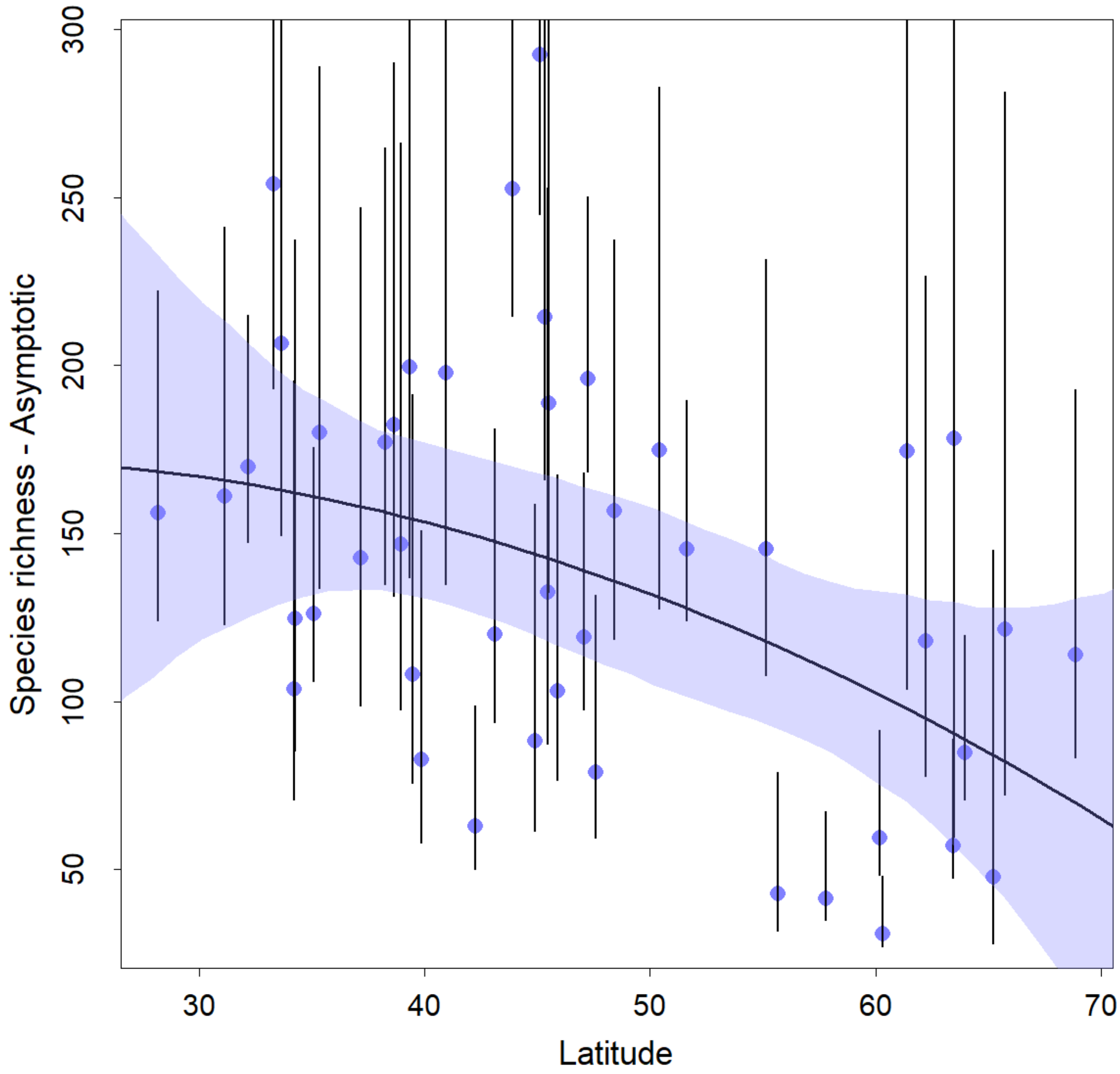

Supplementary Figure 4. Scatterplot and fitted quadratic regression of the relation between the latitude at the centroid and the nonparametric asymptotic estimate of total number of species entire mammal assemblages for each ecoregion included in the analysis. Upper and lower confidence intervals for the diversity estimates are included as bars for each point. 95% credibility interval for the regression models are presented as dashed lines.
